# Electrochemical study of 2-amino-5-mercapto-1,3,4-thiadiazole in the absence and presence of *p*-benzoquinone: an efficient strategy for the electrosynthesis of new 1,3,4-thiadiazole derivatives†

**DOI:** 10.1039/d2ra07250e

**Published:** 2023-01-19

**Authors:** Hossein Masoumi, Sadegh Khazalpour, Mahdi Jamshidi

**Affiliations:** a Faculty of Chemistry, Bu-Ali Sina University Hamedan 65178-38683 Iran Khazalpour@gmail.com

## Abstract

In this study, first, the electrochemical behavior of 2-amino-5-mercapto-1,3,4-thiadiazole (AMT) was fully investigated in the absence and presence of electrochemically generated *p*-benzoquinone (*p*-BQ, which is the oxidized form of hydroquinone), as an electrophile, *via* cyclic voltammetry (CV) at a glassy carbon electrode (GCE) and in an acetic acid buffer (0.2 M)/ethanol solution mixture. Then, an *E*–pH diagram was proposed for different structures of AMT at various pH values. The obtained voltammograms also exhibited an “electron transfer + chemical reaction” (EC) mechanism. Besides the voltammetric exploration, electrosynthesis of new 1,3,4-thiadiazole derivatives was conducted by constant current electrolysis (CCE) as a facile and cost-effective method for the formation of S–S and S–C bonds. Finally, the biological activity of products was also analyzed *via* an *in silico* method.

## Abstract 2

An electrochemical study on the redox behavior of 2-amino-5-mercapto-1,3,4-thiadiazole (AMT) in the absence and presence of *p*-benzoquinone (*p*-BQ) and its application in the electrosynthesis of new 1,3,4-thiadiazole derivatives.

## Graphical abstract

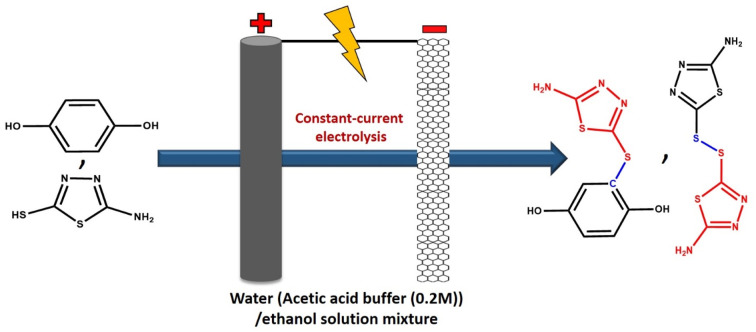

## Introduction

**Scheme 1:**
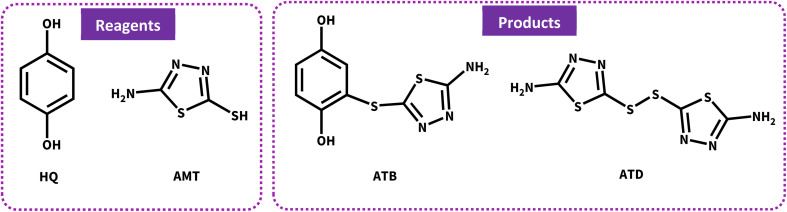
Structure of primary materials and products.

Many synthetic transformations are based on the oxidation number variation, albeit these redox processes usually include short lifetime species, making their investigation difficult. Therefore, in recent decades, electrochemical techniques have drawn much attention because they can facilitate the exploration of these processes.^1–3^ The unique benefits of this redox-modulating technique are in great commensurate with the principles of green chemistry.^4–6^ Some of them can be highlighted as follows: low energy and temperature consumption, high selectivity, good atom economy, and low costs for reagents; moreover, electrons are considered as clean reagents.^5^ They can also be conducted to obtain both kinetic and thermodynamic details of oxidation–reduction reactions.^7^ Among different electrochemical techniques, cyclic voltammetry and constant current electrolysis (CCE) have been developed as significant methods for the quantitative and qualitative characterization of complex processes on the surface of electrodes.^8–10^ Generally, 1,3,4-thiadiazole derivatives are well-known five-membered heterocycles that display a broad spectrum of biological activities such as antimicrobial, anti-inflammatory, antituberculosis, anticonvulsant, and anxiolytic functions.^11,12^ For over a decade, thiadiazole-based molecules have been widely used as drugs because of their special biological activities, but unfortunately, the widespread use of thiadiazoles has led to the development of severe resistance, which significantly reduced their efficacy.^13^ This study was focused on 2-amino-5-mercapto-1,3,4-thiadiazole (AMT). The nucleophilic property of AMT results from two active groups of amine and thiol (NH_2_ and SH); therefore, with these strong electron donor groups, AMT has been used for different applications,^14^ in particular, polymerization reactions.^15^ Furthermore, hydroquinone (HQ) is a renowned component in many natural sources such as fruits and vegetables, and it has many pharmaceutical properties and is widely used as an antioxidant, anticancer, and even anti-hyperpigmentation agent in skin-lightening creams.^16,17^*p*-Benzoquinone (*p*-BQ), which can be electrochemically generated from HQ, is considered a very reactive electrophile, and it can be attacked by various nucleophiles. In this study, the electrochemical behavior of AMT in the absence and presence of *p*-BQ as an electrophile was investigated by CV in different aqueous buffer solutions (pH = 4.00–8.00, *c* = 0.2 M) and pH = 5.0 has been chosen as the optimum media. Due to the impressive properties of both the mentioned species and the demand for molecules with unique properties, electrosynthesis and discovery of new thiadiazole derivatives as future drugs have been performed to develop new effective therapies. Consistent with the significant data displayed by voltammograms, we first involved AMT in a Michael-type addition reaction with *p*-BQ to synthesis 2-((5-amino-1,3,4-thiadiazol-2-yl)thio)benzene-1,4-diol (ATB), and then during our electrochemical study, the oxidized structure of AMT showed its great potential as a radical specie; thus, for the first time ever, we propose a green electrochemical protocol for the production of AMT dimer (Scheme 1), which was chemically synthesized before by different research groups.^18,19^

For medicinal purposes, in the last section of this study, we used an *in silico* method to assess the biological activity of the electrosynthesized compounds. By using this method, the conditions for new 1,3,4-thiadiazole derivatives will be examined to manufacture drugs in the future. In this regard, the interaction of electrochemically generated products as inhibitor ligands was investigated within the active sites of myeloperoxidase (PDB ID: 1DNW), NADPH oxidase (PDB ID: 5VN0), cytochrome P450 3A4 (PDB ID: 4D75), *E. coli* topoisomerase IV (PDB ID: 3FV5) and *Lactobacillus brevis* (PDB ID: 1ZK4).

## Results and discussion

### pH effect on the electrochemical oxidation of AMT

**Fig. 1:**
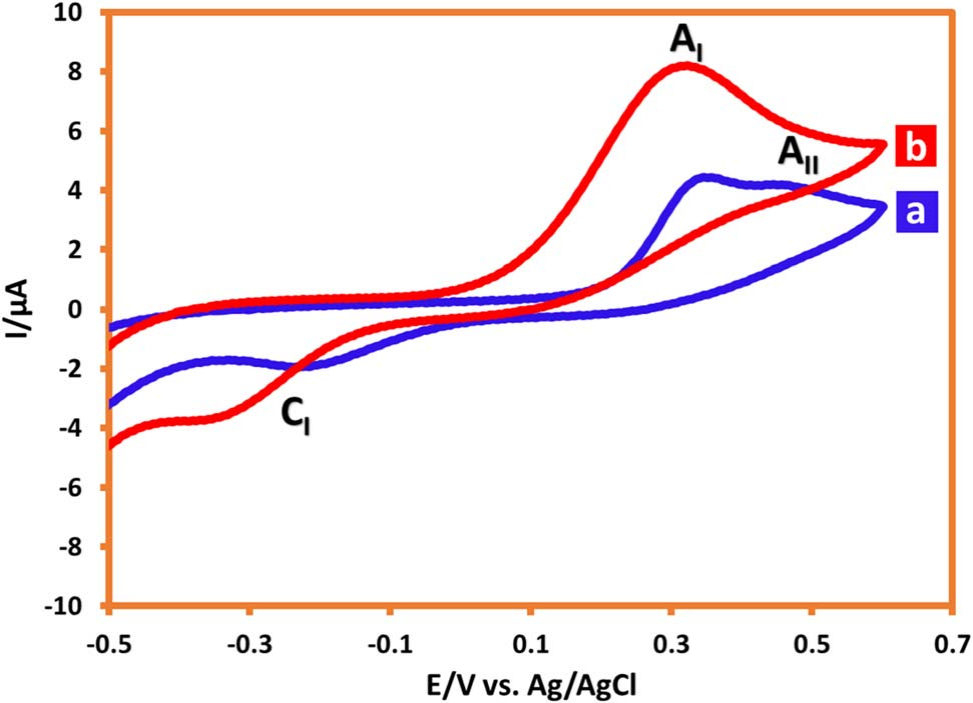
Cyclic voltammograms of 1 mM AMT in (a) ethanol/acetic acid buffer (10/90), and (b) acetonitrile/acetic acid buffer (90/10). Scan rate: 100 mV s^−1^. Room temperature.

Cyclic voltammograms of AMT (1 mM) in ethanol/water (10/90) and acetonitrile/water (90/10) mixtures are depicted in Fig. 1, respectively. According to the mentioned voltammograms, AMT in the ethanol/water mixture exhibits two anodic and one cathodic peaks. The first anodic peak (A_I_) belongs to the oxidation of AMT, the second anodic peak (A_II_) can be due to the adsorption, and the third peak (C_I_) is also attributed to the reduction of AMT_ox_ (oxidized form of AMT) to AMT. In this regard, to verify A_II_ as an adsorption peak, the redox behavior of AMT was also investigated in the acetonitrile/water (90/10) mixture. The obtained CV perfectly implies that A_II_ was an adsorption peak. Herein, according to the obtained result, the effect of pH on the electrochemical oxidation of AMT was considered in an acetonitrile/water mixture to determine the peak potential position clearly; but the investigation of redox behavior and the electrolysis of AMT were both conducted in the ethanol/water mixture because of its green aspect (Fig. 4 and 8).

**Fig. 2:**
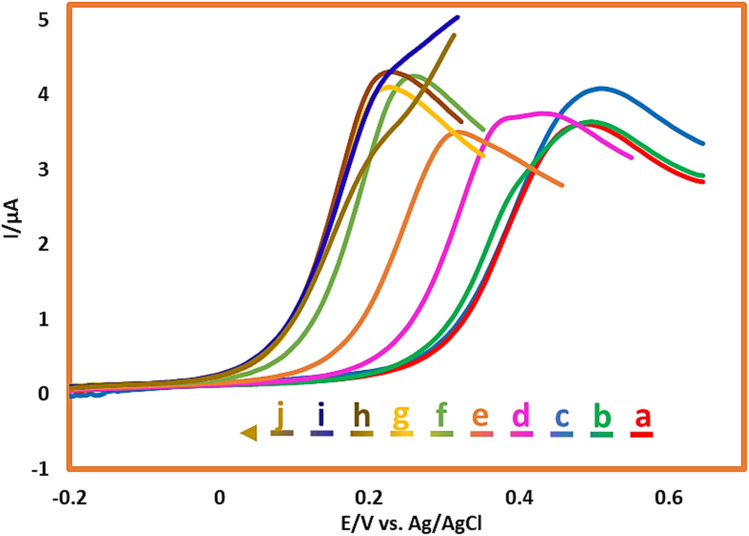
LSVs of AMT (1.0 mM) in a buffer/acetonitrile solution mixture (with different pH values (90/10 v/v)) at GCE. Scan rate: 50 mV s^−1^. Room temperature. pH values from a to j are: 1.0, 2.0, 3.0, 4.0, 5.0, 6.0, 7.0, 8.0, 9.0 and 10.

**Fig. 3:**
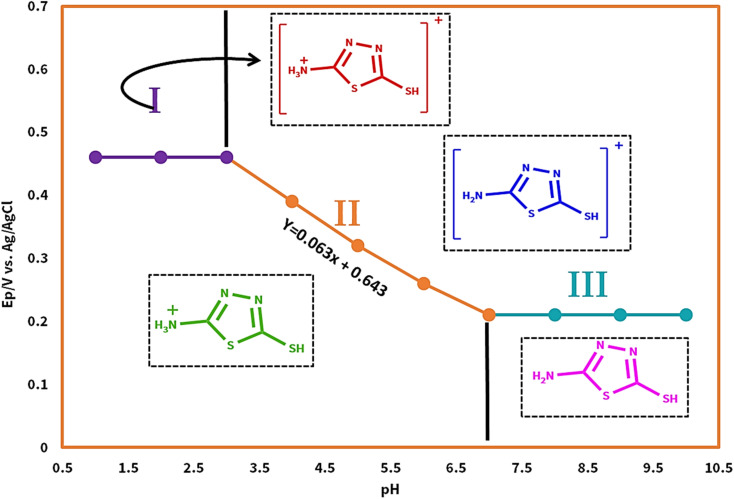
Potential–pH diagram of AMT.

The effect of pH on the electrochemical behavior of AMT (1.0 mM) was studied in different aqueous buffer solutions from pH = 1.0 to pH = 10.0 using the linear sweep voltammetry (LSV) technique. As shown in Fig. 2, the steady increase in the pH value resulted in the movement of peak potential to more negative potentials at a specified region (pH = 3.0 to pH = 7.0). This observation confirms the involvement of H^+^ in the oxidation process of AMT. The following equation will also describe the alteration of anodic peak potential position with pH:*E*_pA_1__ = *E*_p0_ − (2.303*mRT*/2*F*)pHwhere *m* is the number of participating protons in the redox reaction, *E*_p0_ is the anodic peak potential at pH = 0.0, and *R*, *T*, and *F* have their usual meanings. The dependence of the anodic peak potential to the pH value is plotted in Fig. 3. This diagram consists of three lines with the following slopes: 0 mV per pH (line I: purple), 63 mV per pH (line II: orange), and 0 mV per pH (line III: cyan).

As shown in Fig. 3, at pH values less than 3.0, the slope of line I is equal to 0 mV per pH, which obviously shows the independence of the peak potential to the variation in pH, but in the second region of the potential–pH diagram (pH = 3.0 to 7.0), the slope of line II is 63 mV per pH, which is approximately close to the theoretical value of 60 mV per pH for a one electron-one proton redox reaction. The last part of the mentioned diagram (pH > 7.0) also has the same description as part one.

**Scheme 2:**
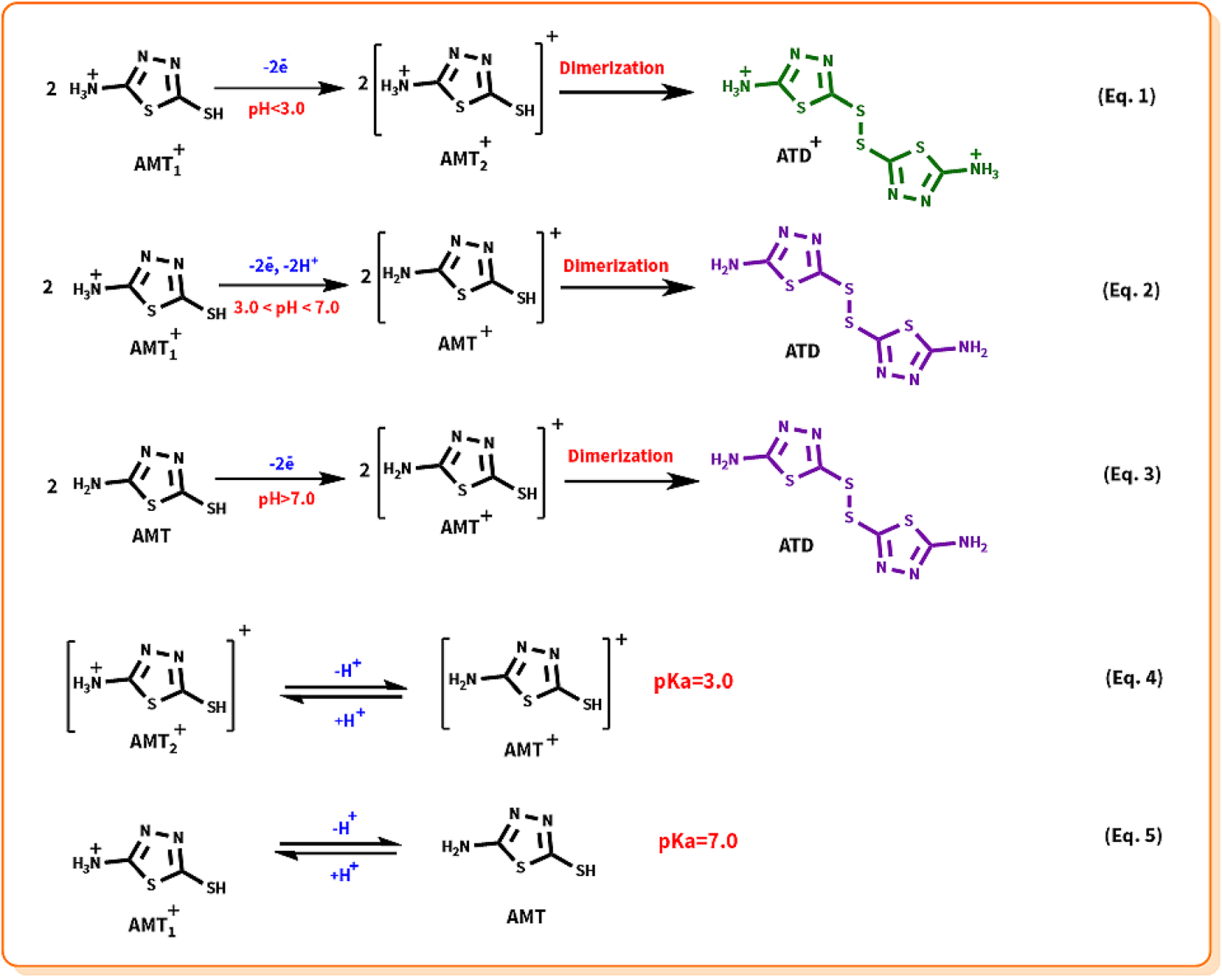
Proposed redox mechanism of AMT at different pH values.

According to the obtained information from the potential–pH diagram and other research group's investigation on the redox behavior of AMT,^20^ we propose a series of mechanisms for the oxidation/reduction process of AMT at different pH values (1.0 to 10.0), and in addition, the p*K*_a_ value of the desired molecule was determined (Scheme 2). In case of our obtained data at pH values less than 3.0, the alternation of peak potential is independent of pH (eqn (1)), and two AMT_1_^+^ molecules (the NH_2_ group of AMT is protonated in this structure) will be oxidized to their cationic states (AMT_2_^+^), and then they will form one AMT dimer (ATD^+^), while for pH values between 3.0 and 7.0, two AMT_1_^+^ molecules must first participate in a two electron–two proton process to generate 2 AMT^+^, and then the result of their reaction will be one AMT-dimer (eqn (2)) with NH_2_ groups (ATD). For pH values greater than 7.0, the AMT was only oxidized to AMT^+^ and no protons were transferred (eqn (3)). Fortunately, as we explain in the further sections, we could synthesize the AMT dimer (ATD), and this achievement helped us to confirm our hypothesized mechanisms.

### Voltammetric and coulometric studies

**Fig. 4:**
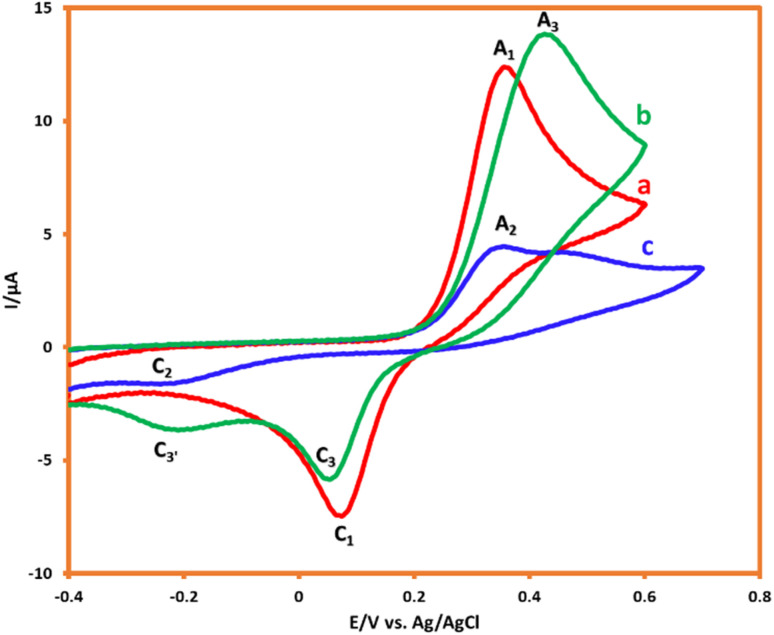
CVs of HQ (1.0 mM) in the (a) absence and (b) presence of AMT (1.0 mM). (c) CV of AMT (1.0 mM). Scan rate: 100 mV s^−1^. Solvent: acetic acid buffer/ethanol mixture (90/10 v/v). Room temperature.

The electrochemical oxidation of HQ (1.0 mM) in the aqueous solution (acetic acid buffer (pH = 5.0)/ethanol mixture (90/10 v/v)) was studied by the CV technique. The resulting cyclic voltammogram displayed the redox couple of A_1_/C_1_ (HQ/BQ), which follows a two-electron, two-proton, reversible reaction (Fig. 4). The electrochemical behavior of AMT (in the absence of HQ) also indicated an irreversible redox system with an anodic (A_2_) and a cathodic (C_2_) peak current. Furthermore, under the same conditions, the electrochemical oxidation of HQ was investigated in the presence of AMT as a nucleophile. As shown in Fig. 4, the cathodic peak current of HQ (C_1_) will decrease and shift to more negative values in the presence of AMT. The decrease in cathodic peak current demonstrates that a chemical reaction occurs in the reverse scan after the electrochemical step.^21^

It is also founded that the peak current ratio of *I*_pC_1__/*I*_pA_1__ depends on the concentration of AMT and the potential scan rate. Therefore, an increase in AMT concentration will decrease the mentioned ratio. In fact, the possibility of a reaction between nucleophiles and electrophiles would be much higher in this way. In a similar fashion, with the increment in potential scan rate, the *I*_pC_1__/*I*_pA_1__ ratio will rise reciprocally, while the current of the cathodic peak will be diminished.^22^ Indeed, the growth of cathodic/anodic peak current ratio at fast scan rates is because the higher scan rates lead to a decrease in the size of the diffusion layer; and consequently, there would not be enough time for AMT to react with *p*-BQ.

**Fig. 5:**
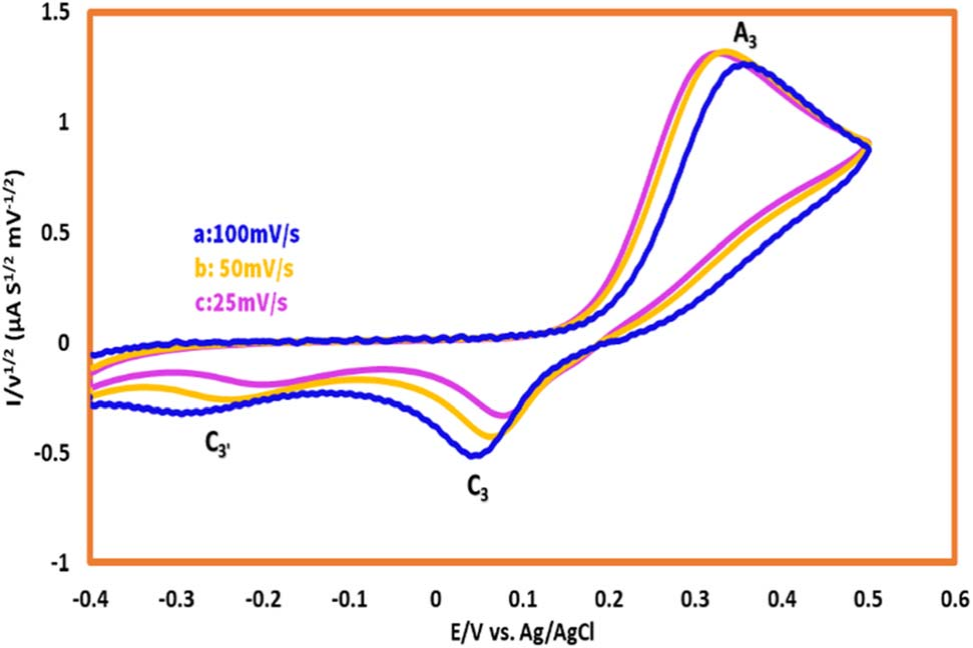
Normalized cyclic voltammograms of 1.0 mM HQ in the presence of 1 mM AMT. Scan rates from a to c are: 100, 50, and 25 mV s^−1^.

According to Fig. 5, the variation in scan rate had controlled the interval of the voltammogram, and this can be compared against the reactivity, therefore to remove the effect of scan rates on CVs, all of the demonstrated voltammograms were normalized by the Nicholson–Shain equation (all the current values had divided to the second root of the scan rate).^23,24^

As can be seen, with the alternation of scan rates from 100 mV s^−1^ to 25 mV s^−1^, the magnitude of cathodic peak (C_3_), which is considered as a factor for the determination of reactivity, will decrease proportionally, and this confirms the reaction between AMT and *p*-BQ. This result indicated that a chemical reaction will occur after the electrochemical step, and this can be interpreted as an EC mechanism.^25^

**Scheme 3:**
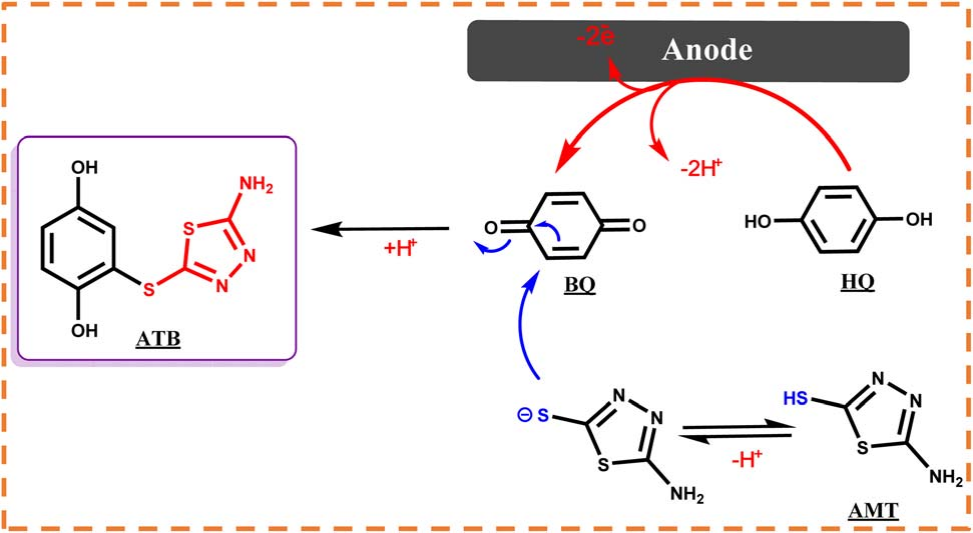
Electrochemical synthesis pathway of ATB.

**Fig. 6:**
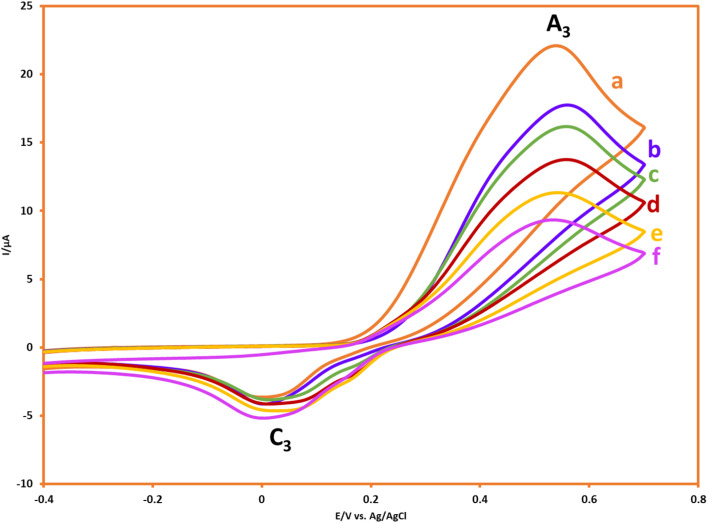
CVs of 0.25 mmol HQ in the presence of 0.5 mmol AMT during CPC at 0.35 V *vs.* Ag/AgCl after consumption of a to f: 0, 10, 20, 30, 40 and 50 coulomb. Scan rate: 50 mV s^−1^. Solvent: acetic acid (0.2 M)/ethanol mixture (50/50 v/v). Room temperature.

In the following, with the insights drawn from the electrochemical behavior of precursors, the controlled-potential coulometry (CPC) was performed at a potential of 0.35 V *vs.* Ag/AgCl reference electrode in the water (acetic acid buffer, 0.2 M)/ethanol mixture (50/50 v/v), which consisted of 0.25 mmol HQ and 0.5 mmol AMT, to determine the exact amount of consumed coulombs and to synthesis a pure product. In the next step, CCE was carried out as a straightforward technique for the optimization of electrolysis (Scheme 3). Cyclic voltammetry was also utilized during the electrolysis process to evaluate coulometry advancement (Fig. 6). It was found that A_3_ will proportionally see a decline with the progress of electrolysis, and the obtained CVs had also shown that the reaction will be ended after consuming two electrons per molecule.

**Fig. 7:**
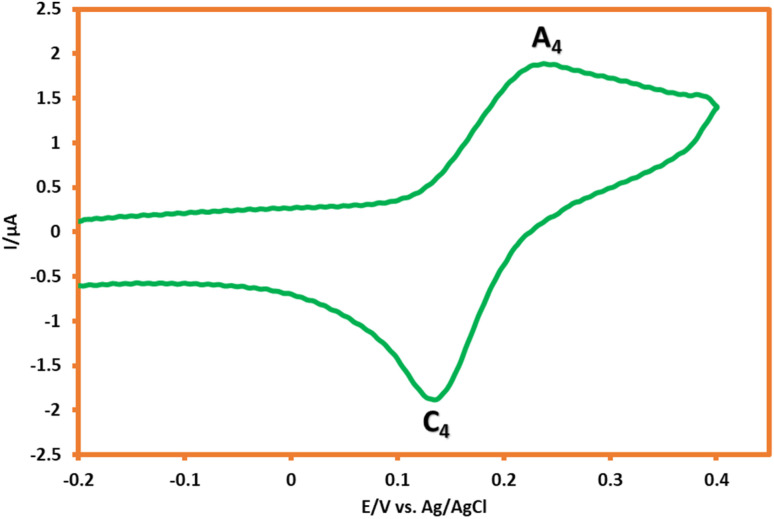
Cyclic voltammogram of the saturated solution of ATB in water (acetic acid buffer, pH = 5.0, *c* = 0.2 M)/ethanol mixture (90/10 v/v). Scan rate: 50 mV s^−1^. Room temperate.

**Scheme 4:**
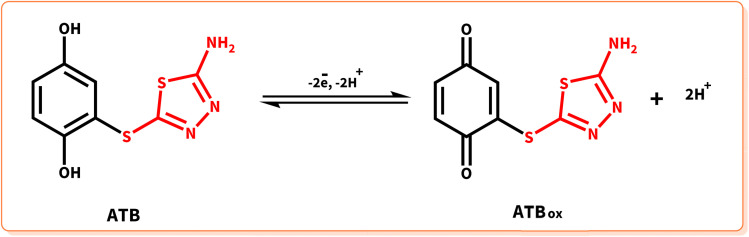
Redox behavior of ATB.

The cyclic voltammogram of the product (ATB) is shown in Fig. 7. According to this CV, the anodic peak potential of the final product (A_4_) shifted to more negative values than HQ's voltammogram. The oxidation/reduction potentials of 0.23 V and 0.13 V (A_4_ and C_4_) *vs.* Ag/AgCl reference electrode for the product can dedicate to the redox couple of ATB and ATB_ox_, respectively (Scheme 4). This phenomenon can perfectly show that the existence of an electron-donating functional group (–NH_2_) in the structure of ATB made the electrochemical oxidation of the product more desirable than its monomers (it is thermodynamically more suitable to occur).

**Scheme 5:**
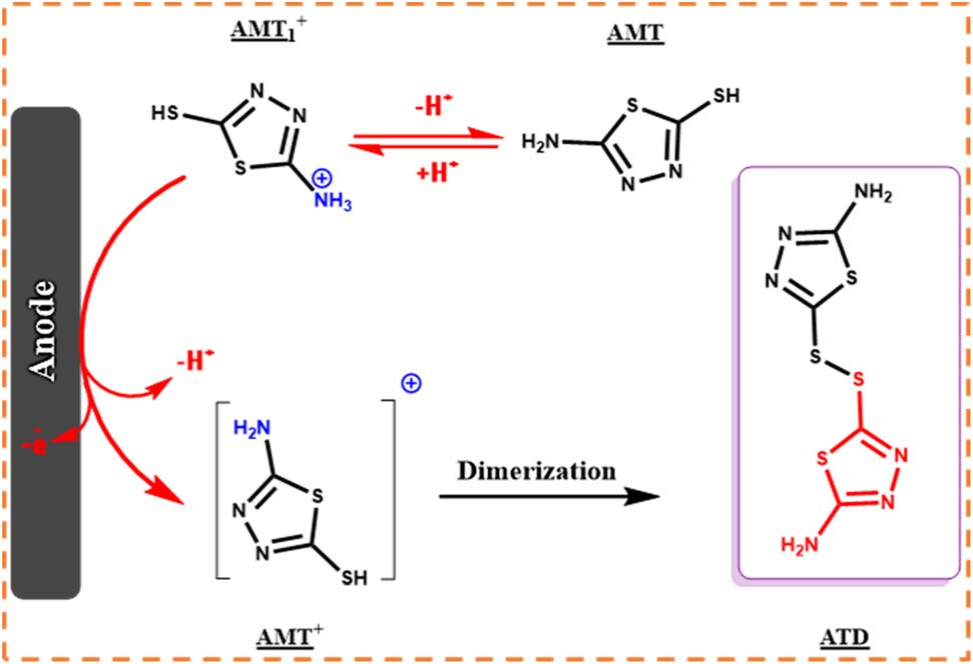
Electrochemical synthesis pathway of ATD.

**Fig. 8:**
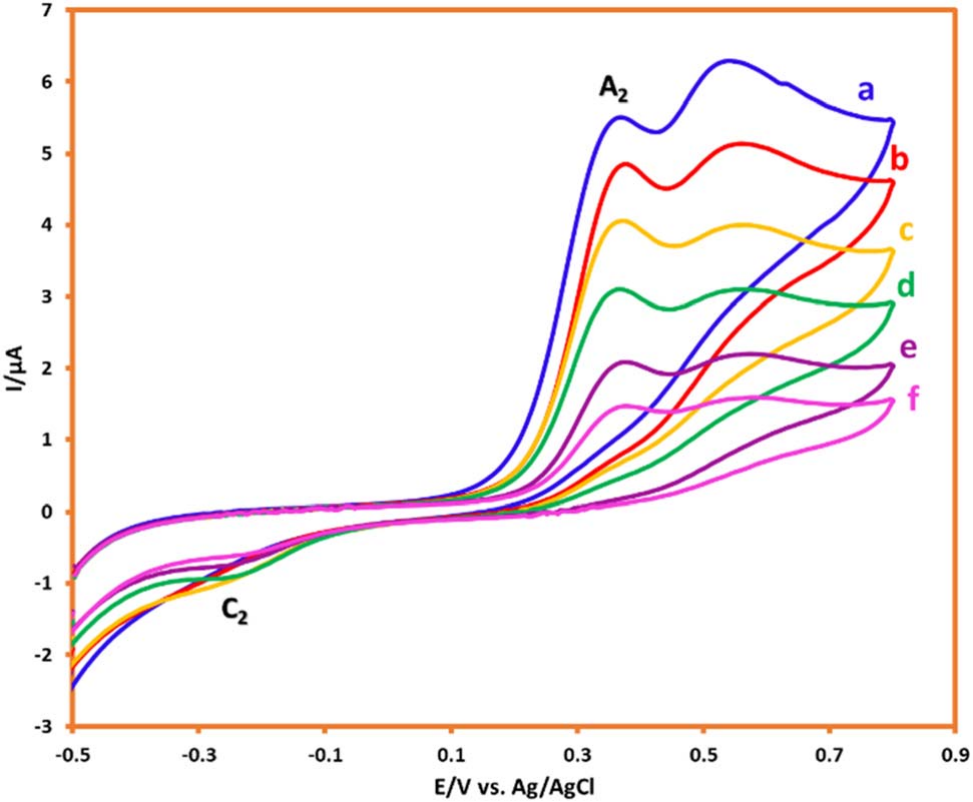
CVs of 0.25 mmol AMT during CPC at 0.38 V *vs.* Ag/AgCl after consumption of a to f: 0, 5, 10, 15, 20 and 25 coulomb. Scan rate: 50 mV s^−1^. Solvent: acetic acid (0.2 M)/ethanol mixture (50/50 v/v). Room temperature.

In the meantime, the formation of a thin yellowish layer on the carbon anodes attracted our attention. On account of this observation, because of the color, solubility, and melting point difference of the yellowish film and gray powder of ATB,^18^ we supposed that this yellowish layer on the surface of anodes might be the AMT dimer or polymer (the idea has come from the high potential of AMT in the polymerization).^26^ Subsequently, the electrolysis process of AMT was also performed individually under similar conditions of ATB synthesis procedure (Scheme 5). The recorded CVs during electrolysis (AMT was converted into AMT_1_^+^ as discussed in Scheme 5*via* protonation reactions) were monitored that first AMT_1_^+^ will be oxidized to AMT^+^ and then AMT^+^ will immediately react with the same specie to form ATD (radical–radical coupling reaction). Herein, the electrolysis process was terminated after consumption of one electron per molecule (Fig. 8). In the following, the result excellently verified the electrochemical dimerization theory of AMT, but in contrast to ATB synthesis, this time AMT was used as an active radical in the radical–radical coupling reaction instead of reacting as a nucleophile. Based on the mentioned data, we present a new and green approach for the electrochemical dimerization of AMT for the first time ever. The utilization of green solvents (water/ethanol mixture) instead of toxic solutions, electrons as clean and economic pre-reagents, time-efficiency, high yield, and pure product are just some of the superiorities of this method in comparison with the conventional chemical pathways for the dimerization of AMT.

### Reaction pathway

In the first step, the anodic oxidation of hydroquinone will generate the corresponding *p*-BQ intermediate, which contains four equivalent Michael acceptor carbons, and then *p*-BQ will be following a pattern of an EC mechanism in the reaction with deprotonated AMT to synthesis ATB; however, alongside this reaction, the electrochemical dimerization of AMT will occur simultaneously on the surface of anodes and results in the formation of a yellowish film of ATD on the mentioned electrodes. The electrochemical synthesis of ATD was also individually carried out under the optimized conditions, and in this case, ATD was formed in a radical–radical coupling reaction *via* oxidized states of AMT. The successful synthesis of ATD with an acceptable yield over pH = 3.0 has validated our proposed reactions for the dimerization mechanism of AMT as stated in the previous sections.

One of the vantage points of the electrochemical procedures compared with the conventional chemical approaches is various methods and parameters that can be optimized for better results. Therefore, the pH of supporting electrolyte, current density, electrode materials, and type of cell (undivided and divided), type of organic solvent and methods of electrolysis (CCE and CPE) were all optimized as influential variables on the yield and purity of desired products using one-factor-at-a-time (OFAT) technique.^27,28^

### Effect of current density

**Fig. 9:**
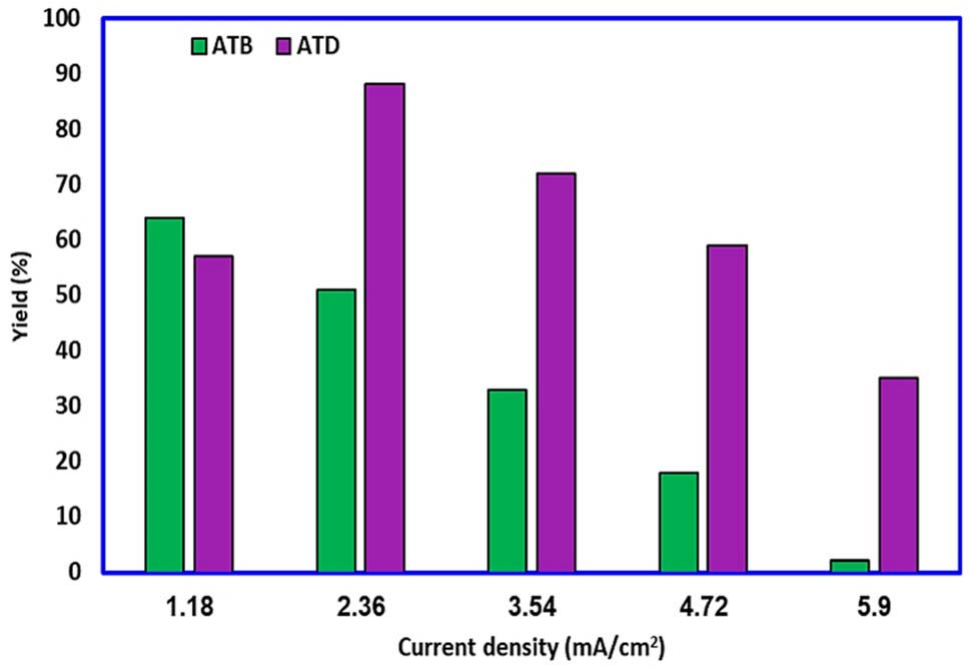
Effect of current density on the yield of ATB and ATD. Amount of HQ: 0.1 mmol. Amount of AMT: 0.2 mmol. Solvent: ethanol/acetic acid buffer (0.2 M) mixture (50/50 v/v). Room temperature.

The electrosynthesis of ATB and ATD was first investigated by the galvanostatic method (because of time frugality and instrumental simplicity). The current density has been changed from 1.18 mA cm^−2^ to 5.90 mA cm^−2^ (0.01–0.05 A). At the same time, other variables (passed charge, concentration of reagents, temperature, *etc.*) remained constant. The result (Fig. 9) indicated that at a current density of 1.18 mA cm^−2^, the highest yield (64%) for ATB will be achieved. It must also be noted that at current densities over the value of 1.18 mA cm^−2^, the possibility of unfavorable reactions such as over-oxidation, dimerization, and polymerization will increase proportionally and then the yield of ATB can decrease. As mentioned previously, during the electrochemical generation of ATB, ATD will also be formed on the surface of anodes as a thin film. Fortunately, the solubility of ATD in an ethanol/buffer mixture is very low; hence, this phenomenon has no impressive effect on the purity of ATB, but the existence of this layer on the surface of electrodes will reduce the active power of cell and the yield of the desired product. Thus, according to the depicted graph (Fig. 9), ATD has a low yield at 1.18 mA cm^−2^ but a high yield at 2.36 mA cm^−2^ current densities.

### Effect of electrolyte solution pH

**Fig. 10:**
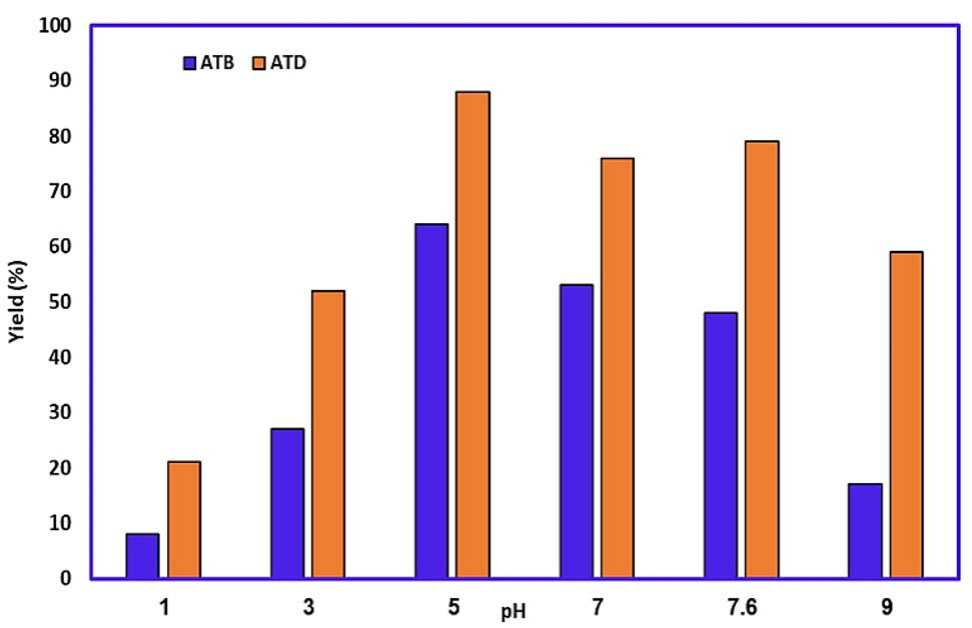
Effect of pH on the yield of ATB and ATD. Amount of HQ: 0.1 mmol. Amount of AMT: 0.2 mmol. Solvent: ethanol/buffer solutions with different pH mixture (50/50, v/v). Current density for ATB: 1.18 mA cm^−2^, current density for ATD: 2.36 mA cm^−2^. Room temperature.

The effect of buffer solution pH on the yield of products was also studied using constant current electrolysis, and results are summarized in Fig. 10. It is evidenced that the highest yield of ATB and ATD was obtained under mild acidic and neutral conditions, respectively (all other parameters have been kept constant as well as before). As mentioned, the highest yield for ATB (64%) and ATD (88%) was achieved at pH = 5.0. With the increase in pH to more amounts, the yield of both reactions will be decreasing gradually. The concentration of the desired deprotonated form of AMT (thiol deprotonation) will increase over pH = 3.0, thereby the possibility of a reaction between AMT and *p*-BQ has been increased; however, with the soar of pH value the oxidation of AMT would be much easier too, and the possibility of polymerization and dimerization reactions of AMT will also have increased proportionally. In this way (the possibility of *p*-BQ hydroxylation will also rise in basic media), the mentioned phenomenon can result in the drop of ATB's yield over pH = 5.0.

### Effect of anodic materials and cell type

The anodic material has significant importance on the yield of electrochemical reactions among all other influential variables. Consequently, carbon and stainless steel were utilized as economic and accessible electrodes. The highest yield for ATB and ATD (64% and 88%, respectively) was achieved by using flat graphites as the anode after passing 2 and 1 F mol^−1^ of electricity in order. The yield of reaction decreased (<5%) when stainless steel was used as the anode. Hereafter, the effects of other parameters were investigated by using the carbon plates as the anode.

**Scheme 6:**
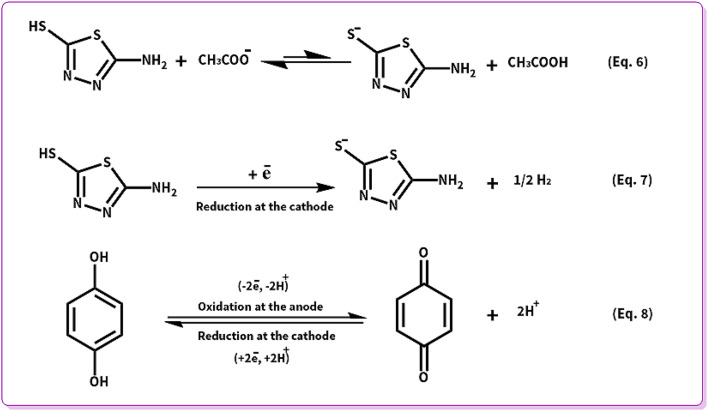
Electrochemical deprotonation and redox pathways of AMT and HQ.

In the next step, the cell type has been changed as an effective factor on the yield of products. Henceforth, the electrolysis processes were performed in the divided and undivided cells. According to Scheme 6, AMT can behave as a strong nucleophile when it is deprotonated (AMT_de_) in contrast to its usual form. Thereby, the high concentrations of AMT_de_ are necessary for the electrosynthesis of ATB. Deprotonation pathways of AMT are demonstrated in eqn (6) and (7).

According to eqn (6) and (7), the highest yield for ATD (88%) will be attained when all the mentioned reactions (eqn (6) and (7)) occur in an undivided cell. While for ATB, the best yield (64%) will be achieved in a divided cell, and the reason is indicated in eqn (8), as can be seen when there is no separator between the anode and the cathode; *p*-BQ after generation at the surface of the anode will immediately reduce at the surface of the cathode to regenerate HQ. Consequently, the lower concentration of *p*-BQ was existing to react with AMT_de_ and *vice versa*. Ergo, the yield of ATB will have decreased in an undivided cell (17%).

### Effect of organic solvents

Organic molecules usually have very low solubility in water;^29^ thus, for the coulometry experiments, a mixture of water and organic solvent was utilized to enhance the solubility. Therefore, types of organic solvents were optimized for this purpose. At first, ethanol was used as a green solvent. In this case, ATB and ATD were synthesized with yields of 64% and 88%, respectively; however, in this manner, the adsorption of AMT on the carbon electrodes is intense, and a large amount of ethanol was used for the electrosynthesis of products (since AMT will not easily dissolve in the common organic solvents, at least 50 mL ethanol is required to dissolve 0.5 mmol of AMT, while the solution should also be placed in an ultrasonic bath). The formation of ATD as a layer on the surface of graphite electrodes will reduce the conductivity of anodes and accordingly will increase the ohmic resistance of the electrochemical cell. In the next level, acetonitrile (at least 35 mL for dissolving AMT) was used as a co-solvent of buffer solution, and this organic solvent can decrease the adsorption rate of ATD on the surface of anodes, and due to this phenomenon, the refreshment of electrodes is not necessary as before, and the IR drop will not increase impressively too (hence the consumption of energy decreased), but the yield of reaction will diminish from 64% to 56% at the best-optimized mode in compression of using ethanol. However, the solubility of AMT and ATB in acetonitrile is much better than that in ethanol; still, the separation of products and residual nucleophile would be difficult in this manner (chromatography techniques are required to separate ATB, ATD, and AMT from each other) and acetonitrile is not a green solvent too.

### Molecular docking studies

In today's world, many scholars have been started using theoretical studies to guide and support experimental research. In this regard, theoretical methods can help us reduce the duration and cost of empirical studies and achieve optimum results.^30–32^ Therefore, in this section of research, we had used computer docking calculations as a powerful tool for the elucidation of the potential of ATB and ATD as future antioxidant and antibacterial drugs. In this theoretical part, the interaction of electrosynthesized compounds as inhibitor ligands were investigated with the active regions of antioxidant target receptors; myeloperoxidase (PDB ID: 1DNW), NADPH oxidase (PDB ID: 5VN0), cytochrome P450 3A4 (PDB ID: 4D75) and antibacterial proteins; *E. coli* topoisomerase IV (PDB ID: 3FV5) and *Lactobacillus brevis* (PDB ID: 1ZK4). It must also be noted that before performing a molecular docking study, some work should be done to prepare the target proteins as follows:^33^ (1) all the water molecules of crystal structures should be removed. (2) All the existing cofactors, and inhibitor ligands within the structure of receptors should be removed. (3) The polar hydrogen atoms should be added to the residues. After the preparation of the protein structure, the docking process of ATB and ATD was conducted using the Molegro Virtual Docker (MVD) software.^34^ As a result of the calculations, the numerical values of the binding affinity (kJ mol^−1^) have been used to compare the inhibition activity of ligands against the above-mentioned proteins. In this regard, many other parameters were also obtained, among which the most significant parameter is the docking score.^10^ The more negative numerical value of this parameter demonstrates the better biological activity of the electrosynthesized molecules against the target proteins. For simplification, we only reported the highest MolDock Score (Table 1) in the main text.

**Table 1:** Binding affinity (kJ mol^−1^) of docking results for the electrosynthesized compounds

Compounds	4d75	1dnw	5vn0	3FV5	1ZK4
ATB	−100.22	−112.21	**−113.43**	−98.77	**−106.57**
ATD	−101.75	−98.09	**−120.05**	**−105.75**	−105.57

**Fig. 11:**
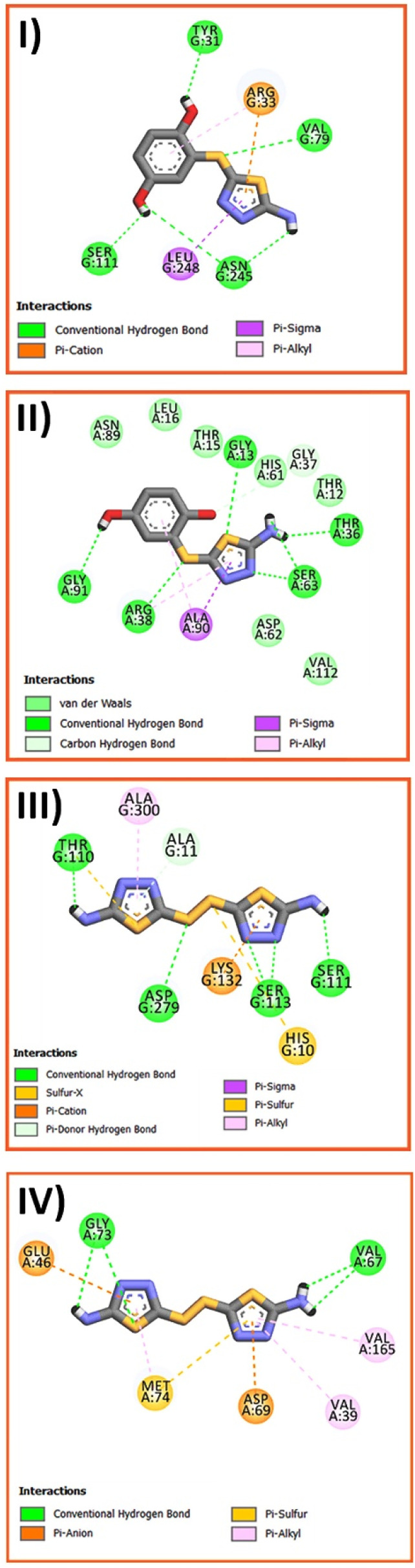
Two-dimensional interactions between ATB and amino acid residue of: (I) 5vn0 (II) 1ZK4 and ATD with (III) 5vn0 and (IV) 3FV5.

According to Fig. 11, conventional hydrogen bonding, van der Waals, Pi-cation, Pi-anion, Pi-donor hydrogen, Pi-sigma, Pi-alkyl, and Pi-sulfur are a variety of interactions that formed among different moieties of ATB and ATD with amino acid residues of target proteins. Here, we only investigated the strongest ligand–receptor interactions to simplify results (more detailed information can be found in the ESI†). The results reported in Table 1 indicate that both ATB and ATD have the strongest interactions (−113.43 and −120.05 kJ mol^−1^) with active regions of 5vn0 among other reactive oxygen species (ROS) generated. The visualized interactions in Fig. 11 depicted that important conventional hydrogen bonds of ATB were constructed between H atoms of hydroxyl groups and TYR 31, SER 111, ASN 245, H atom of amine group and ASN 245 and finally the bridged sulfur atom of two rings with VAL 79. However, for ATD, the conventional hydrogen bonds were formed between the H atom of amine groups and THR 110, SER 111, nitrogen atom of thiadiazole ring and SER 113, and finally, the bridged sulfur atom of two rings with ASP 279. Furthermore, ATB and ATD interacted strongly with amino acid residues of 1ZK4 and 3FV5 respectively, as antibacterial target proteins. In this case, most important interactions also included the conventional hydrogen bonds.

## Experimental

### Apparatus and reagents

Cyclic voltammetry was performed using a Metrohm Autolab model PGSTAT 204 potentiostat/galvanostat. Controlled-potential coulometry (CPC) and constant current coulometry (CCC) were performed using a Behpazhoh model 2051 potentiostat/galvanostat. In the voltammetry experiments, a glassy carbon disc (1.8 mm diameter), a platinum wire, and a Ag/AgCl electrode were utilized as the working, auxiliary, and reference electrodes, respectively (all electrodes from AZAR Electrode Co.). Three carbon plates (6 × 1.4 × 0.05 cm) were used as the anode, and a stainless steel mesh grid was selected as the cathode in the electrolysis process. Hydroquinone and 2-amino-5-mercapto-1,3,4-thiadiazole were both provided from commercial sources and used without further purification.

### Electroorganic synthesis of 2-((5-amino-1,3,4-thiadiazol-2-yl)thio)benzene-1,4-diol (ATB)

**Scheme 7:**
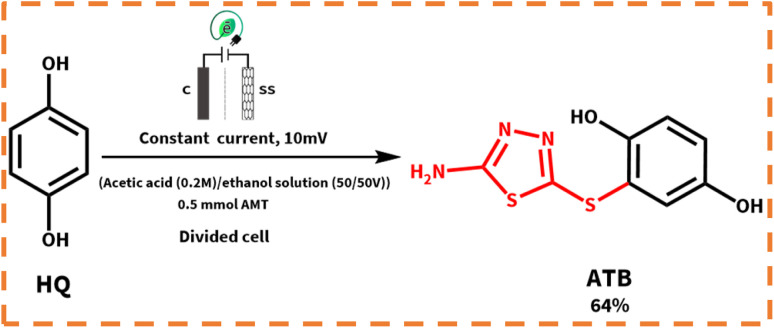
Electrochemical procedure for the synthesis of ATB.

At first, 0.25 mmol HQ and 0.5 mmol AMT were dissolved in a 100 mL flask filled with 50 mL ethanol and 50 mL acetic acid buffer (0.2 M, pH = 5.0), and then the solution mixture was electrolyzed in a divided cell at 0.35 V *vs.* Ag/AgCl (Scheme 7). Immediately after passing 20 min of the electrolysis process, a thin layer will form on the surface of carbon electrodes because of drastic adsorption of AMT; due to this phenomenon, all the graphite electrodes were constantly washed with acetone to be refreshed during synthesis (every 10 coulomb by stopping the electrolysis). The coulometry was instantly stopped after consumption of 50 coulomb (in this regard, the electrolysis process should be stopped to prevent undesired reactions such as over oxidation and dimerization). After electrolysis, a Rotavapor was used to vaporize the organic phase of the solution, and then the residual water phase was acidified with dilute HCl and then extracted with ethyl acetate. The separated organic layer was dried using a Na_2_SO_4_ salt, filtered, and evaporated. The precipitated powder was collected and then washed several times with distilled water. In the next level, after drying, the obtained gray solid was also washed with 10 mL ethyl acetate (this step was done to remove the impurities from the product). Eventually, the structure of the product was perfectly recognized *via* FTIR spectroscopy, ^1^H NMR, ^13^C NMR, and MS.

Isolated yield: 64%. Mp: 189–192 °C (Dec.) ^1^H NMR (300 MHz, methanol-d_4_) *δ* 6.80 (d, *J* = 3 Hz, 1H, aromatic), 6.77 (s, 1H, aromatic), 6.73 (d, *J* = 3 Hz, 1H, aromatic), 6.62 (s, 2H, NH_2_). ^13^C NMR (75 MHz, methanol-d_4_) *δ* 180.51, 173.08, 151.82, 151.32, 120.69, 119.41, 118.03, 116.82. IR (KBr): *ν* 3435, 3288, 3177, 1559, 1413 cm^−1^. MS (El, 70 eV): *m*/*z* (relative intensity%): 241.2 (1.73), 149.1 (8.39), 133 (7.12), 110.1 (6.81), 97.1 (22.4), 71.1 (33.6), 69.1 (79.9), 51.1 (71.8), 43.1 (100).

### Electroorganic synthesis of bis-(5-amino-1,3,4-thiadiazol-2-yl)disulfide (ATD)

**Scheme 8:**
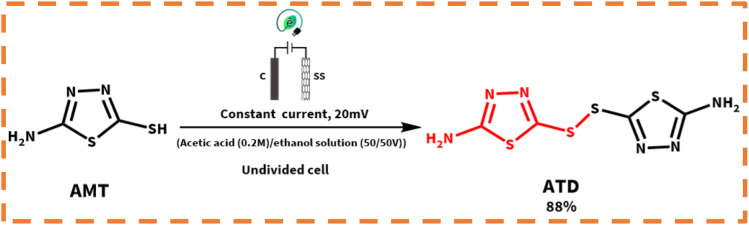
Electrochemical procedure for the synthesis of ATD.

In the first step, 0.25 mmol AMT was dissolved in a 100 mL beaker-type undivided cell, which was filled with the same solvents as for ATB at 0.38 V *vs.* Ag/AgCl (Scheme 8). When the coulometry was end-up, a yellow solid was obtained, filtrated and then washed several times with distilled water. The resulting product was then dried and identified by spectroscopic methods, which were used for ATB.

Isolated yield: 88%. Mp: 235–237 °C. ^1^H NMR (300 MHz, DMSO-d_6_) 7.75 (4H, NH_2_). ^13^C NMR.^15^ IR (KBr): *ν* 3261, 3088, 1631, 1631, 1321, 1136 cm^−1^. MS (El, 70 eV): *m*/*z* (relative intensity%): 264.3 (3.8), 221 (4.4), 135 (11.1), 133 (100), 83.1 (12.5), 74.1 (31.3), 69.1 (17.4), 57.1 (79.4), 43.1 (47.8).

### Molecular modeling

The crystal structures of antioxidant target proteins myeloperoxidase (PDB ID: 1DNW), NADPH oxidase (PDB ID: 5VN0), cytochrome P450 3A4 (PDB ID: 4D75), and antibacterial proteins of *E. coli* topoisomerase IV (PDB ID: 3FV5) and *Lactobacillus brevis* (PDB ID: 1ZK4) were all extracted from the online Protein Data Bank (PDB: https://www.rcsb.org). The ligand's structure was first drawn using the ChemBio Ultra software (version: 16.0, Cambridge Soft). Then, their geometry optimization was performed using the AVOGADRO software. In this study, Molegro Virtual Docker (MVD) was used for the simulation process, and the visualization of obtained results was performed using the discovery studio software. The parameter setting of docking wizard options was also set as follows: score function: MolDock Score, ligand evaluation: internal ES (internal HBond, sp^2^–sp^2^ torsions and were all marked), number of runs: 30 runs, docking algorithm: MolDock SE, max. steps: 300; max. population size: 50, neighbor distance factor: 1.00, maximum iterations: 1500, max. The number of poses returned: 30.

## Conclusion

In summary, this work presented the rapid, efficient, and straightforward electrosynthesis of ATB and ATD with acceptable yields of 64% and 88%, respectively under the conditions which are following principles of green chemistry (no use of toxic solvents, room temperature, high energy efficiency, *etc.*). For the fulfillment of our purpose, the redox behavior of AMT has been explored in the absence and presence of *p*-BQ at various pH values by cyclic voltammetry. The achieved information approved that a chemical reaction will immediately occur after the electrochemical oxidation of HQ or AMT (the reaction follows an EC mechanism). In another exploration, the potential pH diagram of AMT was also drawn, and different structures of AMT in every pH were proposed for the first time ever. In the following, a series of experiments have been performed to optimize the effect of various parameters on the yield and purity of desired products. In the end, we had used the molecular docking method as an efficient tool for the assessment of biological activity (antioxidant and antibacterial activity) of the electrosynthesized compounds. The results of docking, revealed that both ATB and ATD binds strongly to active sites of different antioxidant and antibacterial target proteins. The obtained results from this section also approved that the new electrosynthesized derivatives of 1,3,4-thiadiazole molecules have high potential of being new candidates for the design of novel drugs to treating some diseases, which can be caused by oxidative stress or bacteria.

## Conflicts of interest

There are no conflicts to declare.

## Supplementary Material

RA-013-D2RA07250E-s001

RA-013-D2RA07250E-s002
