# Supplementary material for: Electrochemical study of 2-amino-5-mercapto-1,3,4-thiadiazole in the absence and presence of p-benzoquinone: an efficient strategy for the electrosynthesis of new 1,3,4-thiadiazole derivatives
Source: RSC Adv. 2023 Jan 19;13(5):3083–94. doi: 10.1039/d2ra07250e (PMC9850745; doi:10.1039/d2ra07250e)
Supplement: RA-013-D2RA07250E-s001 [file RA-013-D2RA07250E-s001.pdf]

Electronic Supplementary Material (ESI) for RSC Advances.
This journal is © The Royal Society of Chemistry 2023

Electrochemical study of 2-amino-5-mercapto-1,3,4-thiadiazole in the absence and presence of p-benzoquinone: An efficient strategy for the electrosynthesis of new 1,3,4-thiadiazole derivatives

Hossein Masoumi, Sadegh Khazalpour* and Mahdi Jamshidi

Faculty of Chemistry, Bu-Ali Sina University, Hamedan 65178-38683, Iran

*E-mail: S.khazalpour@basu.ac.ir & Khazalpour@gmail.com ; Fax: +98-813-8257407

No **Page**

1 Spectroscopic characterization of products …………………………………….……………… 3

2 FT-IR spectrum of **ATB** …………………………..……………………………………. 4

3 ^{1}H NMR spectrum of **ATB** ……………………………………..…………………… 5

4 ^{13}C NMR spectrum of **ATB** ……………………………..……………………………... 6

5 Mass spectrum of **ATB** ……………………………………………………………....… 7

6 FT-IR spectrum of **ATD** ……………………………………………………...……… 8

7 ^{1}H NMR spectrum of **ATD** ……………………………………………………………..…… 9

8 Mass spectrum of **ATD** ……………………………………...………………………… 10

9 Molecular modelling of **ATD** and **ATB** ……………………………..…………………… 11

Characterization of products

*Spectroscopic characteristics of 2-((5-amino-1,3,4-thiadiazol-2-yl)thio)benzene-1,4-diol (**ATB)***

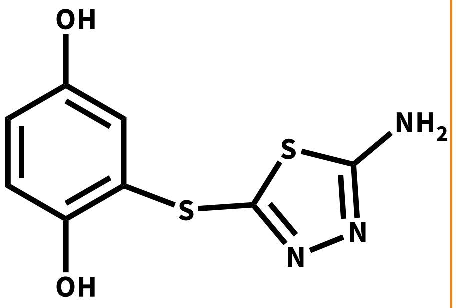

ATB

Isolated yield: 64%. Mp: 189-192°C (Dec.) ^{1}H NMR (300 MHz, Methanol-d4) δ 6.80 (d, J = 3 Hz, 1H, aromatic), 6.77 (s, 1H, aromatic), 6.73 (d, J = 3 Hz, 1H, aromatic), 6.62 (s, 2H, NH2). ^{13}C NMR (75 MHz, Methanol-d4) δ 180.51, 173.08, 151.82, 151.32, 120.69, 119.41, 118.03, 116.82. IR (KBr): ν 3435, 3288, 3177, 1559, 1413 cm−1. MS (El, 70 eV): m/z (relative intensity %): 241.2 (1.73), 149.1 (8.39), 133 (7.12), 110.1 (6.81), 97.1 (22.4), 71.1 (33.6), 69.1 (79.9), 51.1 (71.8), 43.1 (100).

*Spectroscopic characteristics of of Bis-(5-amino-1,3,4-thiadiazol-2-yl) Disulfide (**ATD)***

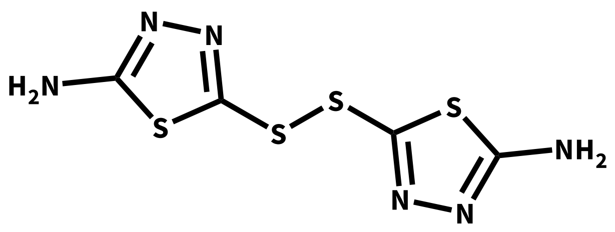

ATD

Isolated yield: 88%. Mp: 235-237°C. ^{1}H NMR (300 MHz, DMSO-d6) 7.75 (4H, S, NH2). ^{13}C NMR [14]. IR (KBr): ν 3261, 3088, 1631, 1631, 1321, 1136 cm−1. MS (El, 70 eV): m/z (relative intensity %): 264.3 (3.8), 221 (4.4), 135 (11.1), 133 (100), 83.1 (12.5), 74.1 (31.3), 69.1 (17.4), 57.1 (79.4), 43.1 (47.8).

FT-IR spectrum of **ATB**

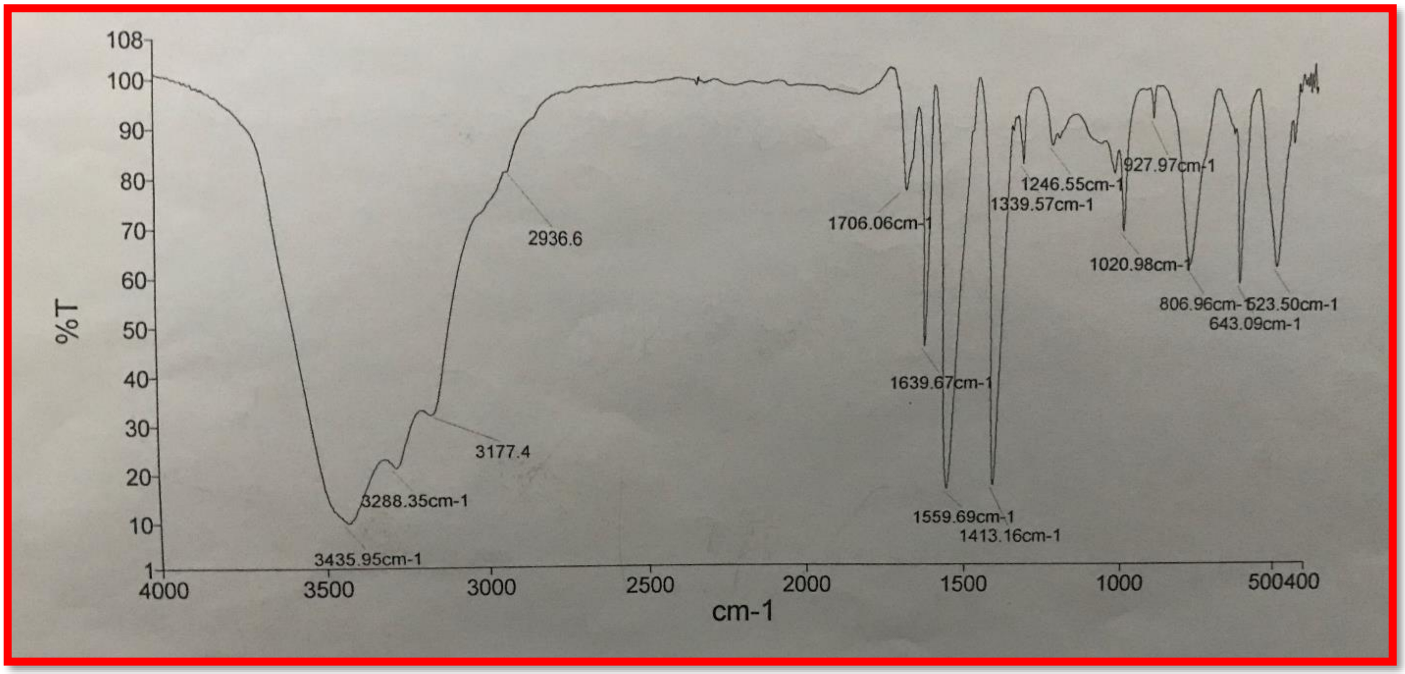

^{1}H NMR spectrum of **ATB**

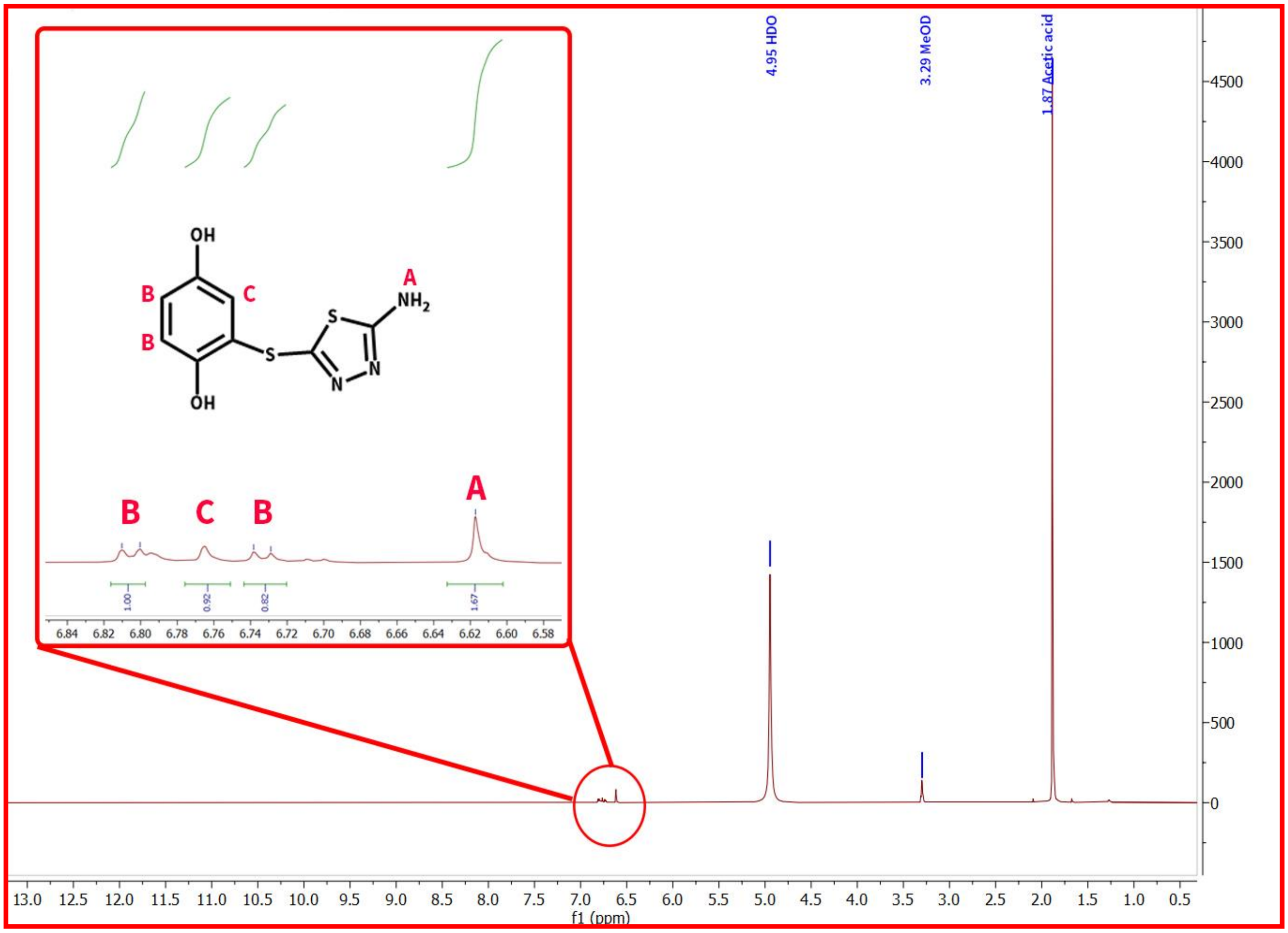

^{13}C NMR spectrum of **ATB**

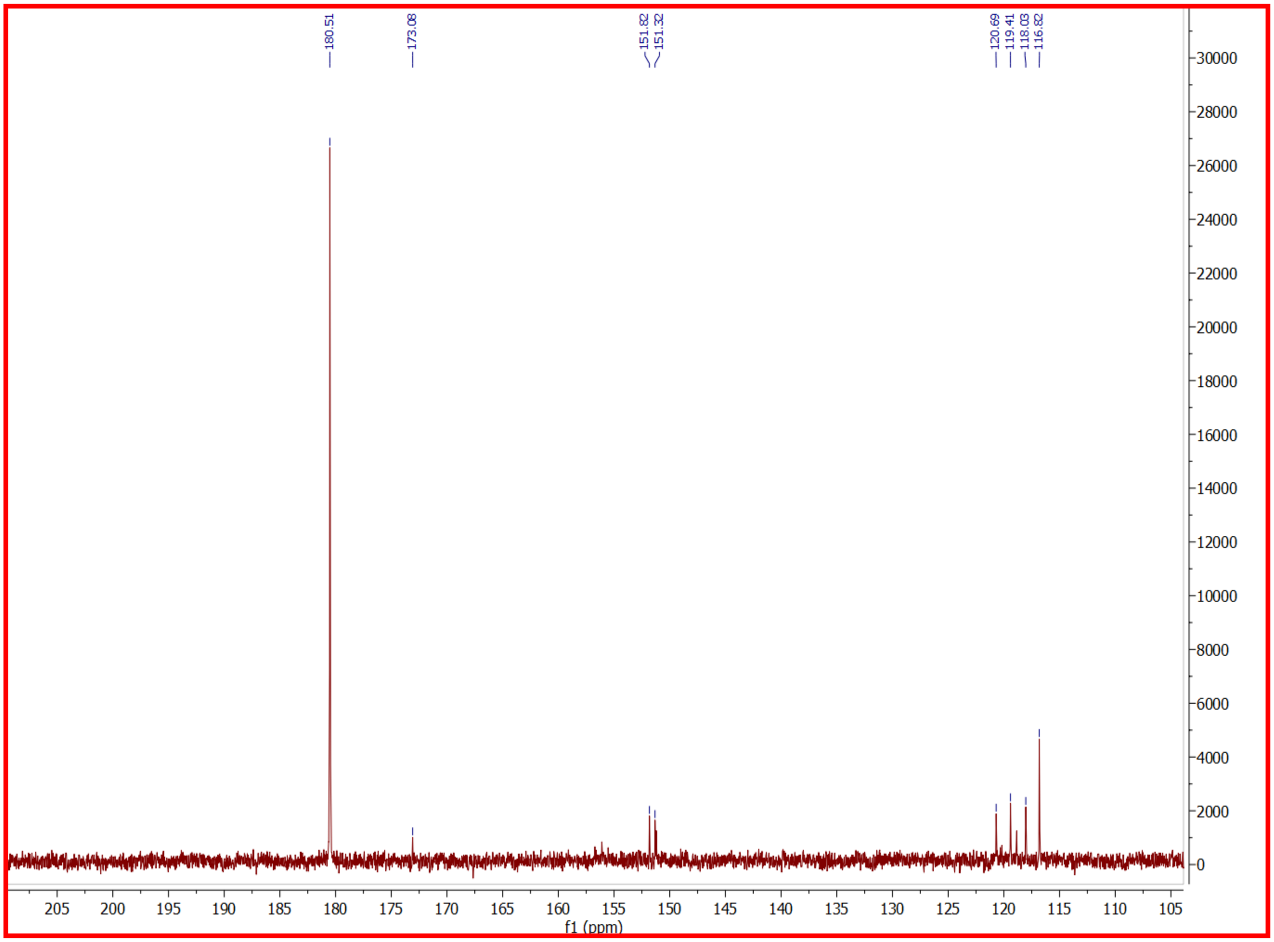

Mass spectrum of **ATB**

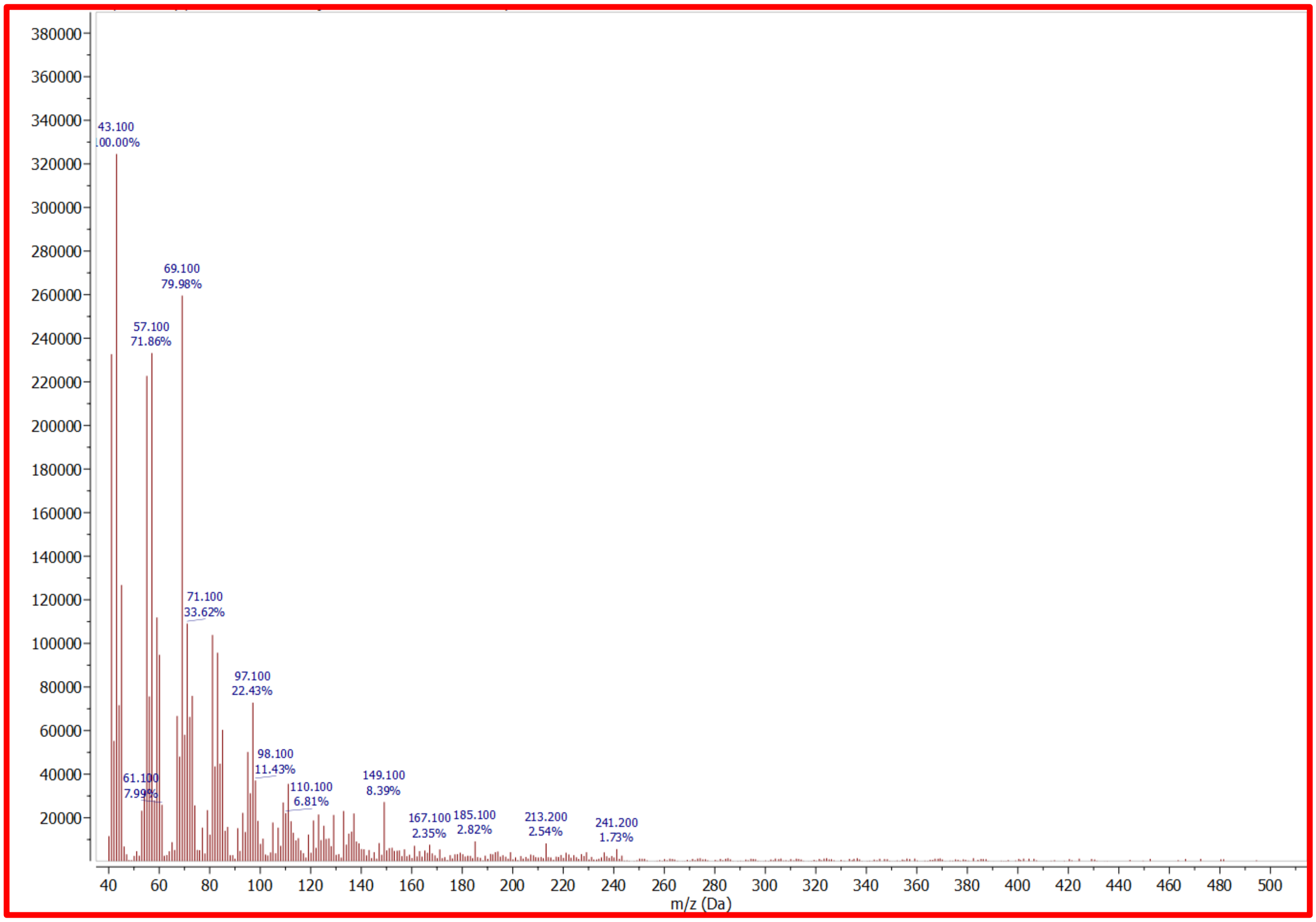

FT-IR spectrum of **ATD**

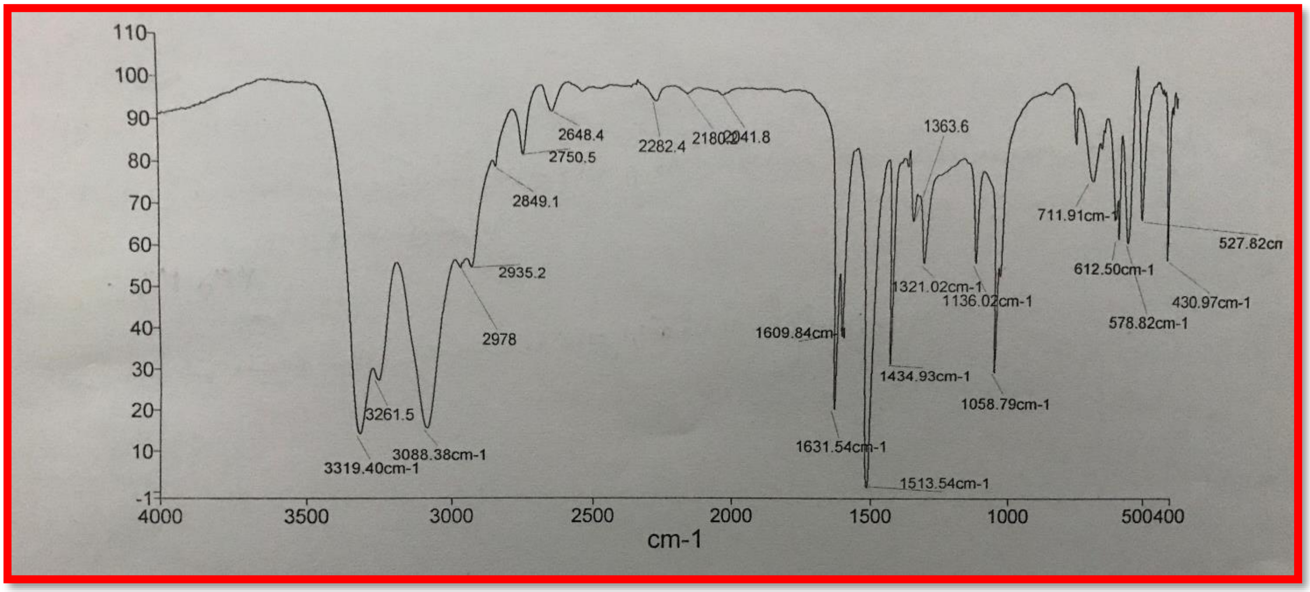

^{1}H NMR spectrum of **ATD**

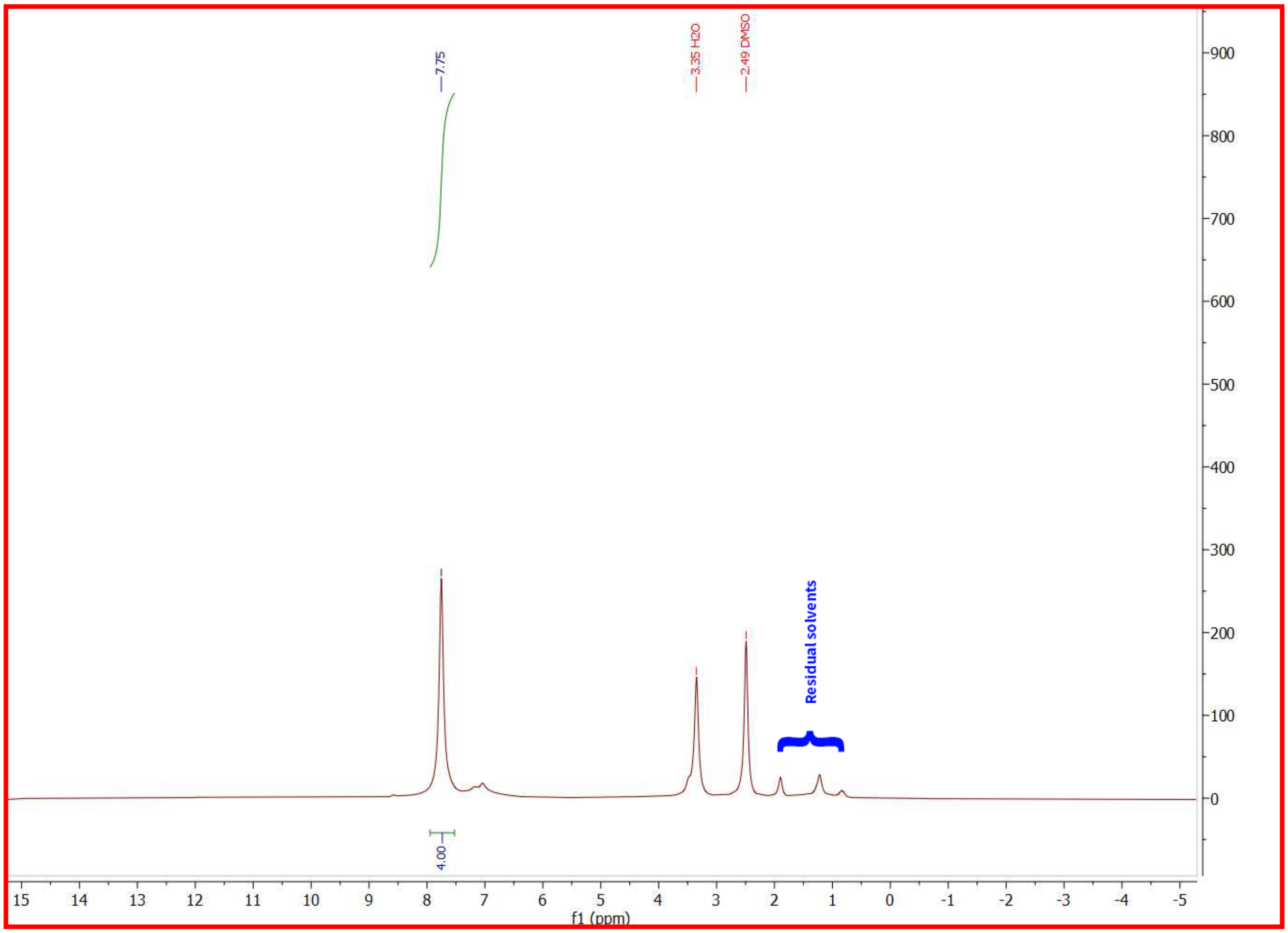

Mass spectrum of ATD

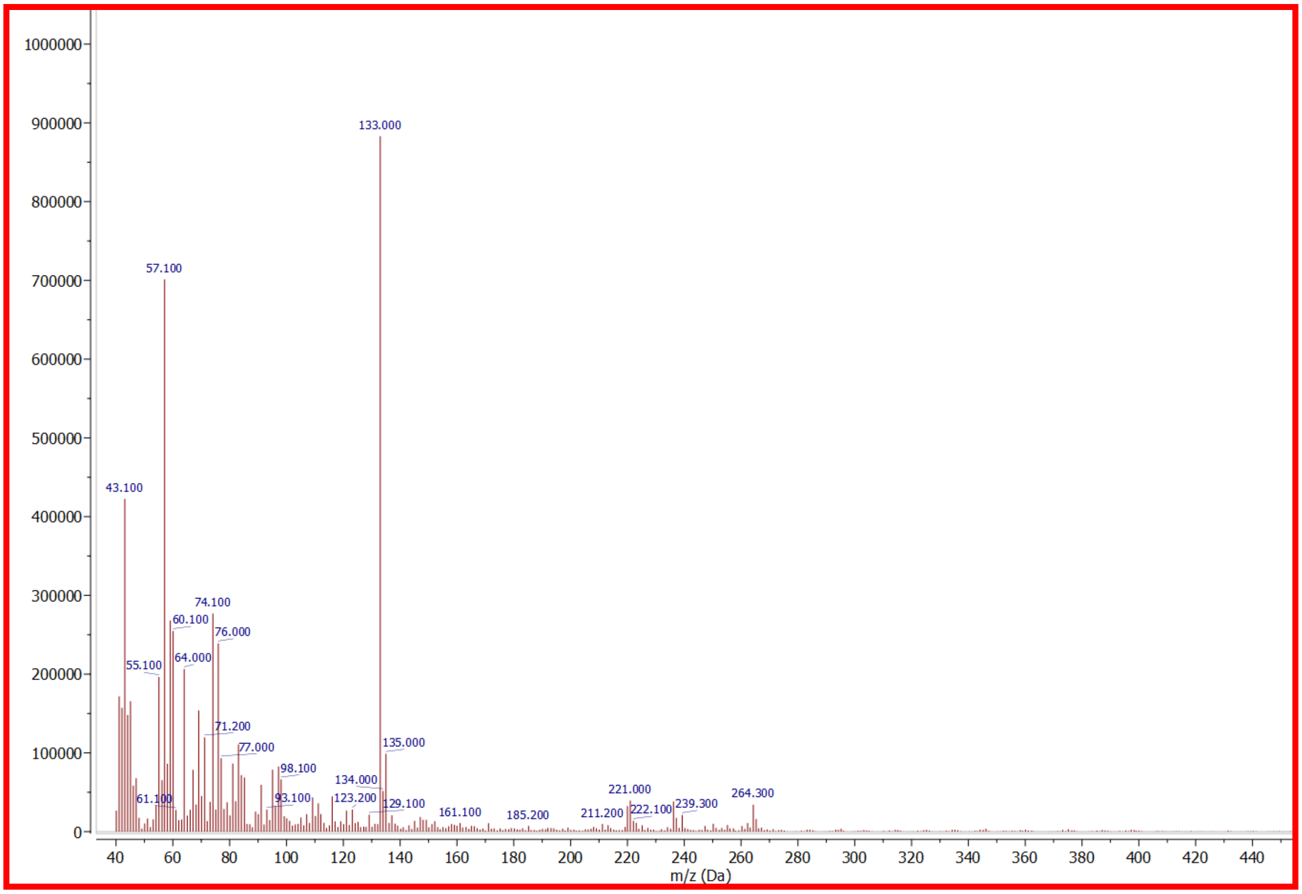

Molecular docking of ATB

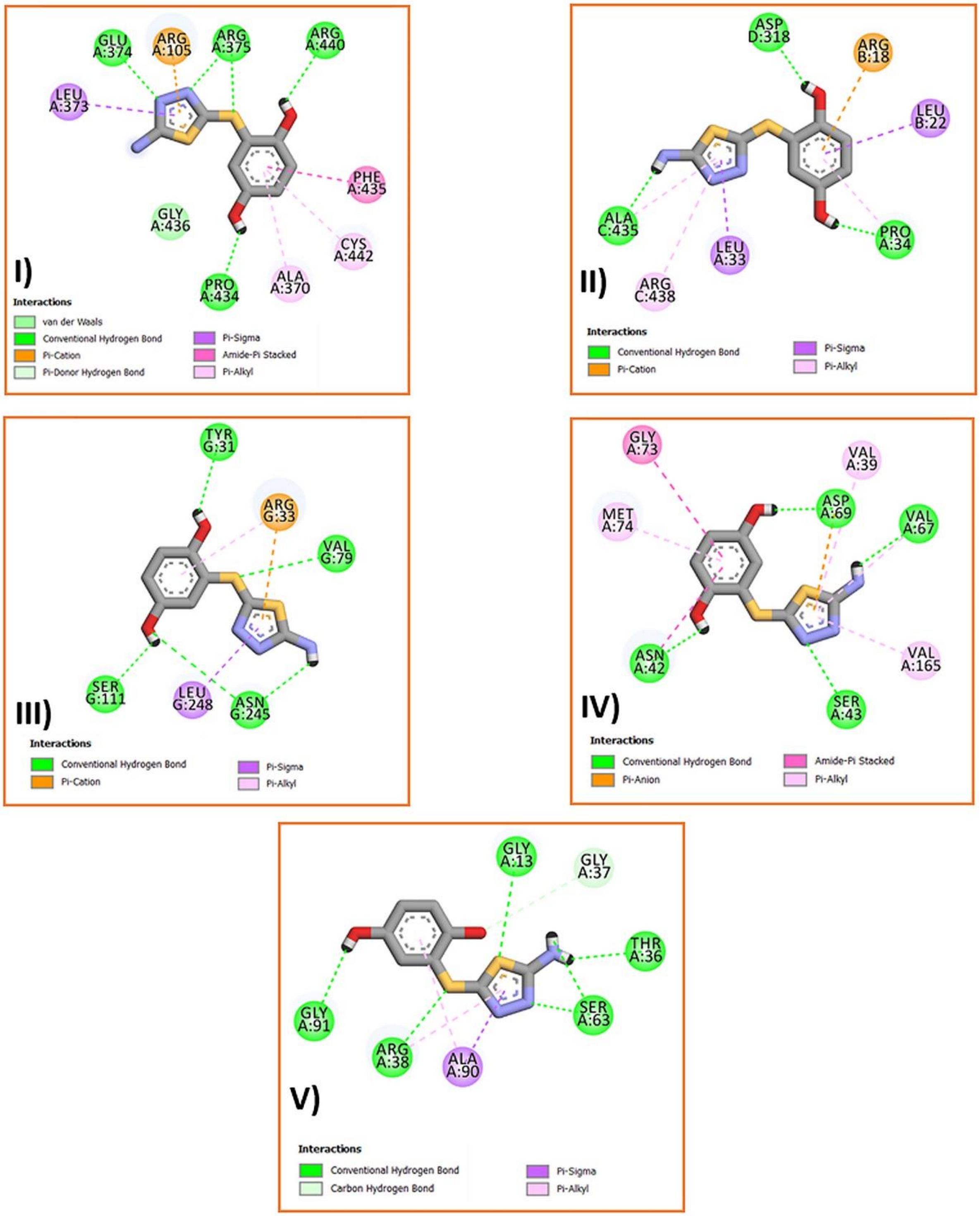

Fig. 1S. 2D interactions between ATB and amino acid residue of:

I) 4d75 II) 1dnw III) 5vn0 IV) 3FV5 V) 1ZK4.

Molecular docking of ATD

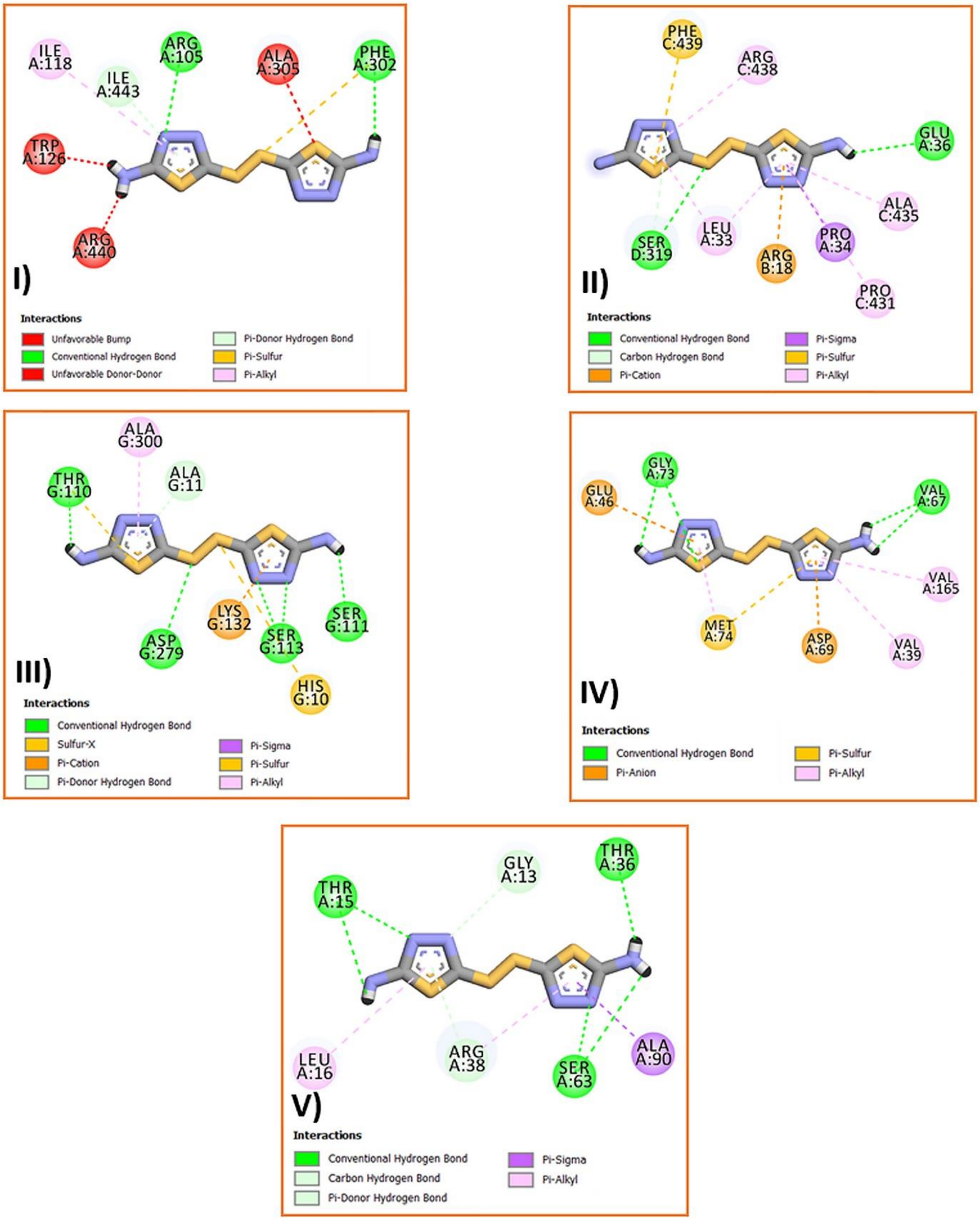

Fig. 2S. 2D interactions between ATD and amino acid residue of:

I) 4d75 II) 1dnw III) 5vn0 IV) 3FV5 V) 1ZK4
